# Melioidosis Patient Survival Correlates With Strong IFN-γ Secreting T Cell Responses Against Hcp1 and TssM

**DOI:** 10.3389/fimmu.2021.698303

**Published:** 2021-07-30

**Authors:** Sineenart Sengyee, Atchara Yarasai, Rachan Janon, Chumpol Morakot, Orawan Ottiwet, Lindsey K. Schmidt, T. Eoin West, Mary N. Burtnick, Narisara Chantratita, Paul J. Brett

**Affiliations:** ^1^Department of Microbiology and Immunology, Faculty of Tropical Medicine, Mahidol University, Bangkok, Thailand; ^2^Department of Medicine, Mukdahan Hospital, Mukdahan, Thailand; ^3^Department of Microbiology and Immunology, University of Nevada, Reno School of Medicine, Reno, NV, United States; ^4^Division of Pulmonary, Critical Care & Sleep Medicine, Harborview Medical Center, University of Washington, Seattle, WA, United States; ^5^International Respiratory and Severe Illness Center, University of Washington, Seattle, WA, United States; ^6^Mahidol-Oxford Tropical Medicine Research Unit, Faculty of Tropical Medicine, Mahidol University, Bangkok, Thailand

**Keywords:** *Burkholderia pseudomallei*, melioidosis, T cells, IFN-gamma, Hcp1, TssM, immune responses, survival

## Abstract

Melioidosis, caused by the Gram-negative bacterium *Burkholderia pseudomallei*, is a serious infectious disease with diverse clinical manifestations. The morbidity and mortality of melioidosis is high in Southeast Asia and no licensed vaccines currently exist. This study was aimed at evaluating human cellular and humoral immune responses in Thai adults against four melioidosis vaccine candidate antigens. Blood samples from 91 melioidosis patients and 100 healthy donors from northeast Thailand were examined for immune responses against *B. pseudomallei* Hcp1, AhpC, TssM and LolC using a variety of cellular and humoral immune assays including IFN-γ ELISpot assays, flow cytometry and ELISA. PHA and a CPI peptide pool were also used as control stimuli in the ELISpot assays. Hcp1 and TssM stimulated strong IFN-γ secreting T cell responses in acute melioidosis patients which correlated with survival. High IFN-γ secreting CD4^+^ T cell responses were observed during acute melioidosis. Interestingly, while T cell responses of melioidosis patients against the CPI peptide pool were low at the time of enrollment, the levels increased to the same as in healthy donors by day 28. Although high IgG levels against Hcp1 and AhpC were detected in acute melioidosis patients, no significant differences between survivors and non-survivors were observed. Collectively, these studies help to further our understanding of immunity against disease following natural exposure of humans to *B. pseudomallei* as well as provide important insights for the selection of candidate antigens for use in the development of safe and effective melioidosis subunit vaccines.

## Introduction

Melioidosis, a serious infectious disease caused by the Gram-negative bacterium *Burkholderia pseudomallei*, is endemic throughout Southeast Asia, South Asia and northern Australia. The clinical symptoms of melioidosis are diverse and range from skin abscesses to acute pneumonia and septicemia. The bacterium can be transmitted to humans through inhalation, percutaneous inoculation and ingestion (1). In 2015, the estimated total global burden of disease was ~165,000 human melioidosis cases with ~89,000 deaths (2). In Thailand and other tropical countries, the mortality rate of melioidosis ranges from 40-50% (3). Most melioidosis patients present with one or more risk factors such as diabetes mellitus, alcoholism, chronic pulmonary disease, chronic kidney disease and thalassemia (4, 5). Since *B. pseudomallei* is intrinsically resistant to many antibiotics, treatment of melioidosis can be challenging. Recurrent melioidosis can occur due to relapse following antibiotic therapy or re-infection with different *B. pseudomallei* strains (6). The ability of *B. pseudomallei* to survive inside non-phagocytic and phagocytic cells also complicates treatment (7). At present, *B. pseudomallei* is classified as a tier 1 select agent by the U.S. Centers for Disease Control and Prevention (8), and there are no vaccines currently available for immunization against melioidosis.

Previous studies have shown that *B. pseudomallei* expresses a variety of conserved protective antigens. These antigens include both polysaccharides (e.g., 6-deoxyheptan capsular polysaccharide (CPS) and lipopolysaccharide) and protein antigens (e.g., BimA, AhpC, LolC, OmpA, OmpW, FliC, TssM and Hcp1) (9–14). *B. pseudomallei* is an intracellular pathogen, thus an effective vaccine will likely require stimulation of both humoral and cellular immune responses to control infections. Several vaccine formulations including live-attenuated, whole-cell killed, subunit, glycoconjugate and outer membrane vesicles have been evaluated and shown to provide various levels of protection in animal models of melioidosis (15–22). These studies suggest that antigen-specific interferon-γ (IFN-γ) secreting T cell responses and serum IgG responses correlate with survival against *B. pseudomallei* infections (15–19, 21, 22), and thus, understanding these protective immune responses could be key for vaccine development.

We and others have previously shown that *B. pseudomallei* can activate several innate immune mediators in whole blood samples from healthy donors (23) and that melioidosis patients have elevated levels of many cytokines including IFN-γ (24, 25). IFN-γ can be produced from peripheral blood mononuclear cells (PBMC) isolated from melioidosis patients when activated with several *B. pseudomallei* antigens *ex vivo.* These antigens include ABC transporter proteins (LolC, OppA and PotF), alkyl hydroperoxide reductase C (AhpC), and a Type III secreted protein (BopE) (21, 26, 27). Two studies in Ubon Ratchathani, Thailand reported the association of survival in melioidosis patients and the number of IFN-γ producing cells in PBMC preparations in response to AhpC activation (21, 26). Another study in Khon Kaen, Thailand demonstrated that patients who recovered from melioidosis had a high number of IFN-γ producing cells that recognized whole bacteria and purified proteins LolC, OppA and PotF (27). Based on these studies, LolC and AhpC are considered to be promising vaccine candidates. Recent studies from our laboratory have shown that proteins associated with the cluster 1 Type VI secretion system (T6SS) such as hemolysin co-regulated protein 1 (Hcp1) and the deubitiquinase TssM can induce strong humoral immune responses in animal models. In addition, when combined with a CPS-CRM197 glycoconjugate, these two proteins provide high-level protection against acute inhalational melioidosis (15). Further studies in humans reported high antibody levels against Hcp1 in melioidosis patients in Thailand and demonstrated that an Hcp1 enzyme-linked immunosorbent assay (ELISA) is a useful serological screening test for use in non-endemic areas as well as endemic areas such as Thailand, Myanmar and Cambodia (28–31). To date, T cell responses in humans against Hcp1 and TssM have not been investigated during acute melioidosis. The characterization and comparison of immune responses against these two candidate vaccine antigens in melioidosis patients and healthy individuals from endemic areas will provide useful insights for development of effective melioidosis vaccines.

This study aimed to investigate the levels and dynamics of cellular and humoral immune responses against four recombinant antigens of *B. pseudomallei* (Hcp1, AhpC, TssM and LolC) in melioidosis patients and healthy individuals from an endemic area. Our study was conducted in Mukdahan province in northeast Thailand where we enrolled 91 culture-confirmed melioidosis cases and 100 healthy donors. We used IFN-*γ* enzyme-linked immunosorbent spot (ELISpot) assays to analyze cellular responses and ELISA to analyze antibody responses in survivors and non-survivors. We further characterized IFN-γ producing cells using flow cytometry. Cellular and humoral immune responses were also monitored in patients who survived melioidosis during a 28-day follow-up period.

## Materials and Methods

### Ethics Statement

Human studies and consent forms were approved by the Ethics committees of the Faculty of Tropical Medicine, Mahidol University (approval number MUTM 2015-002-005 and MUTM 2018-039-02), Mukdahan Hospital (approval number MEC 10/59 and MEC 07/61) and the U.S. Army Medical Research and Development Command, Office of Research Protections, Human Research Protection Office (approval numbers A-20848.a and A-20848.b). The study was conducted according to the principles of the Declaration of Helsinki (2008) and the International Conference on Harmonization (ICH) Good Clinical Practice (GCP) guidelines. Written informed consent was obtained from all patients and healthy donors enrolled in the study.

### Study Design and Subjects

A prospective study of human immune responses to melioidosis was conducted in 91 adult melioidosis patients and 100 healthy donors at Mukdahan Hospital, Mukdahan, Thailand during May 2019 - February 2020. Inclusion criteria for melioidosis patients were male or female of age ≥18 years, admitted to the hospital, culture positive for *B. pseudomallei* within 24 hours and written informed consent was obtained. Exclusion criteria were pregnancy, receiving palliative care or incarceration. Inclusion criteria of healthy donors were male or female of age ≥18 years and written informed consent was obtained. Exclusion criteria were pregnancy or delivery in the past nine months, weight of less than 40 kg or greater than 136 kg, previous history of melioidosis, recent illness, any chronic medical condition or medications and any organ failure (such as cirrhosis), any immune system deficiency, vaccination within the past six weeks, use of any immune-modifying agents or any anti-inflammatory medications or cell depletion biological agents in the past week, infectious symptoms in the past two weeks, vigorous exercise in the past 24 hours, or alcohol use in the past 24 hours. Blood samples were collected from melioidosis patients within 24 hours of when the culture results were provided and from healthy individuals at the time of enrollment. *B. pseudomallei* was identified by biochemical tests and latex agglutination (32) at the Microbiology Laboratory at the hospital and confirmed by Matrix-Assisted Laser Desorption/Ionization-Time of Flight Mass Spectrometry (MALDI-TOF-MS) as previously described (33). The survival status was determined within 28 days using hospital records and contact by telephone. Of a total 91 melioidosis patients, 16 patients (17.6%) died within 28 days. Healthy control subjects were recruited from the blood donation clinic at Mukdahan Hospital.

### PBMC Isolation

PBMC were isolated from 15 ml of heparinized blood within 3 hours of blood draws by density-gradient centrifugation using 50 ml Sepmate™ tubes (STEMCELL Technologies, Canada). In brief, 13 ml of Lymphoprep (Axis Shield, Oslo, Norway) was preloaded into 50 ml Sepmate™ tubes. Whole blood diluted 1:1 with complete RPMI 1640 medium supplemented with 10% heat-inactivated fetal bovine serum (FBS) (Himedia, Mumbai, India) and 2 mM GlutaMAX™ (Gibco, Invitrogen, CA, USA), was then added to the Sepmate™ tubes. Samples were centrifuged for 15 min at 1200 × g at 25°C. The PBMC layer was then gently removed and added to 25 ml of warm complete RPMI medium. The cell suspension was washed twice with 25 ml of complete RPMI medium with centrifugation at 350 × g for 7 min. The PBMC were then slowly resuspended in FBS containing 10% dimethyl sulfoxide (DMSO) (Sigma, USA) at a final density of 5 x 10^6^ cells/ml and brought to -80°C in a freezing container which ensured a slow drop in temperature. PBMC were subsequently transferred to a liquid nitrogen tank for storage until required for use.

### Expression and Purification of Recombinant Proteins

Four recombinant *B. pseudomallei* proteins (Hcp1, LolC, AhpC and TssM) were expressed and purified in this study. Recombinant DNA techniques were performed as previously described (34). Recombinant Hcp1 with an N-terminal 6×His-Tag, *hcp1* (BPSS1498) was PCR amplified from *B. pseudomallei* K96243 genomic DNA using the Bmhcp1-6HisF and Bmhcp1-R1 primer pair (**Table S1**). The resulting DNA fragment was digested with BsaI and cloned into pBAD/HisA digested with NcoI/HindIII producing plasmid pBADBphcp1. Hcp1 was purified from *E. coli* TOP10 (pBADBphcp1) essentially as previously described (28). For the expression of recombinant LolC with an N-terminal 6×His tag, the predicted non-membrane, non-signal peptide-encoding region of LolC (residues 47 to 273) was cloned into pBAD/HisA. A codon optimized gene fragment of LolC (BPSL2277, 139-819 bp) was synthesized (GenScript, Piscataway, NJ), digested with NcoI and HindIII and cloned into pBAD/HisA similarly digested with NcoI and HindIII to produce pBADBplolC. LolC was purified from *E. coli* TOP10 (pBADBplolC) essentially as previously described (28). For expression of recombinant AhpC with an N-terminal 6×His tag, *ahpC* (BPSL2096) was PCR amplified from *B. pseudomallei* K96243 genomic DNA using the BpahpC-FHis1 and BpahpC-R1 primer pair (**Table S1**). The resulting DNA fragment was digested with BsmBI and cloned into pBAD/HisA digested with NcoI/HindIII producing plasmid pBADBpahpC. AhpC was purified from *E. coli* TOP10 (pBADBpahpC) essentially as previously described (28). Recombinant TssM harboring an N-terminal 6×His tag was purified from *E. coli* TOP10 (pMB1001) as previously described (15). DNA sequencing was performed by ACGT Inc, USA.

### Protein Analysis

SDS-PAGE was used to assess the purity of the recombinant proteins (**Figure S1**). Approximately 3 µg of each protein was loaded onto a 12% Bis-Tris gel (Invitrogen, USA) and then visualized following electrophoresis using SimplyBlue Safe Stain (Invitrogen, USA). Endotoxin was removed from the purified proteins using high-capacity endotoxin removal resin (Pierce, USA). Endotoxin levels were quantitated using a LAL chromogenic endotoxin quantitation kit (Pierce, USA). Protein concentrations were determined using a BCA protein assay kit (Pierce, USA). Proteins were sterilized with a syringe filter (0.45 μm) and stored at 4°C.

### IFN-γ ELISpot Assay

Human cellular responses to protein antigens and peptides were assessed by ex-vivo IFN-γ ELISpot assays using cryopreserved PBMC as previously described (26, 35). The 96-well ELISpot assays were performed using human IFN-γ ImmunoSpot^®^ kits (CTL-HIFNG-1M/10) from Cellular Technology Ltd. (CTL, USA). Briefly, the plates were pre-wetted with 35% ethanol and coated overnight with 80 µl of anti-human IFN-γ antibody at 4°C. The next day, plates were washed once with PBS, then antigens in CTL-test™ medium (CTL, USA) were added at a final concentration of 25 µg/ml. PBMC were added in triplicate to wells at 2 × 10^5^ PBMC/well. The spots were developed after incubation at 37°C, 5% CO_2_ for 24 hours. Spot forming units (SFU) in each well were counted using an Immunospot^®^ S6 Micro analyzer (CTL, USA). Results were expressed as IFN-γ spot forming cells (SFC) per million PBMC. Background levels of cellular responses in unstimulated control wells were subtracted from those measured in antigen-stimulated wells. Phytohaemagglutinin (PHA) and Cytomegalo-, Influenza, and Parainfluenza viruses (CPI) peptide pool (CTL, USA) were used as positive controls at final concentrations of 5 µg/ml to ensure T cell responsiveness.

### ELISA

Fifty microliters of antigens in 0.05 M carbonate buffer (pH 9.6) at a concentration of 2.5 µg/ml for Hcp1, AhpC and TssM were added to wells of MaxiSorp U-bottom 96 well plates (Thermo Scientific, Denmark) and incubated overnight at 4°C. The plates were washed 4 times with 300 µl of phosphate buffered saline (PBS) containing 0.05% Tween-20 using a Hydrospeed washer (TECAN, Männedorf, Switzerland) and then blocked with 200 µl of 5% skim milk in PBS for 2 h at 37°C. Human plasma samples were diluted to 1:250 in 1% bovine serum albumin in 0.05% Tween-20 PBS and 50 µl of the diluted plasma samples were added in duplicate and incubated at room temperature for 1 hour. After washing, HRP-conjugated goat anti-human IgG (Sigma) at 1:6000 was added to the plates, and further incubated at room temperature for 2 hours. The plates were washed and then developed with 50 µl of 3,3′,5,5′-Tetramethylbenzidine (TMB) substrate (Invitrogen, USA) for 15 minutes at room temperature. The reaction was then stopped by adding 50 µl of 1N HCl. The optical density (OD) at 450 nm was read using a Sunrise™ microplate reader (TECAN, Männedorf, Switzerland). Pooled culture-confirmed melioidosis plasma (N = 10) was used as a positive control. Pooled healthy control plasma (N = 10) was used as a negative control. The OD value of a blank containing only reagents was subtracted from all OD values of the test samples.

### Flow Cytometry

PBMC at concentration of 1 x 10^6^ live cells/well were stimulated with 25 µg/ml of Hcp1 or TssM or media alone at 37°C with 5% CO_2_ for 2 hours. Phorbol myristate acetate (PMA) plus ionomycin and CPI peptide pool were used as the controls. Brefeldin A (ebioscience, USA) was added at final dilution of 1:1000 and incubated using the same conditions for 4 hours. PBMC were then washed and resuspended in FACS buffer and incubated for 30 minutes with near-infrared live/dead fixable stain (Invitrogen, USA) and fluorochrome-conjugated antibodies: anti-CD3-PerCP (clone UCHT1; BioLegend) at dilution of 1:100, anti-CD4-V450 (clone L200; Becton Dickinson) at dilution of 1:160, anti-CD8-BV510 (clone RPA-T8; BioLegend) at dilution of 1:200, anti-CD56-VioBrightFITC (clone AF12-7H3; Miltenyi Biotec) at dilution of 1:50 for surface marker analysis of CD4^+^, CD8^+^ T cells and NK cells, respectively. After washing with FACS buffer, cells were fixed with fixation solution (BD Biosciences, eBioscience) at 4°C for 20 minutes and then washed with permeabilization buffer. Intracellular staining was subsequently performed with anti-IFN-γ-PE (clone 4S.B3; BioLegend) at dilution of 1:40 for 30 minutes at 4°C. Finally, the samples were washed twice with permeabilization buffer and then resuspended in 200 µl of FACs buffer. Compensation was performed using CompBeads (BD Biosciences) individually stained with each fluorochrome-conjugated antibody. Samples were analyzed immediately using FACAriaIII (BD) or stored at 4°C in the dark for up to 24 hours prior to data acquisition. Data was analyzed using FlowJo software version 10 (Treestar Inc, USA). Lymphocytes were first identified by a low forward scatter (FSC) and low side scatter (SSC) gate, and excluded doublets using FSC-A *versus* FSC-H profiles and dead cells using SSC-A *versus* fixable near-infrared live-dead profiles. Total IFN-*γ* secreting cells were then identified within the single live cells gate. Subsequent gating was divided into CD3^+^CD4^+^, CD3^+^CD8^+^ and CD3^-^CD56^+^ cells for CD4^+^ T cells, CD8^+^ T cells and NK cells, respectively.

### Statistical Analysis

Statistical analysis was performed using GraphPad Prism version 6 (San Diego, CA, USA). The results between subject groups were compared using the non-parametric Mann-Whitney U-test. Significant differences between time points within a group were determined using the non-parametric paired Wilcoxon test. The relationship between IFN-γ ELISpot and IgG-ELISA was evaluated using Spearman’s rank correlation test. *P* value (2-tailed) of < 0.05 was considered significant.

## Results

### Melioidosis Subjects

We enrolled a total of 91 individuals with culture-confirmed melioidosis during May 2019 - February 2020. Demographic characteristics of individuals with melioidosis are shown in **Table 1**. The median age was 53 years (IQR 45-57 years) and 55/91 (60%) were male. The overall 28-day mortality rate was 17.6% (16/91). The most common underlying medical condition among the patients was diabetes (71/91, 78%).

**Table 1:** Demographic characteristics of individuals with melioidosis.

Demographic Characteristic	All (n = 91)	Survivors (n = 75)	Non-survivors (n = 16)
Age, median (interquartile range)	53 (45-57)	52 (44-57)	57 (50-69)
Male gender (%)	55 (60)	44 (59)	11 (69)
Diabetes (%)	71 (78)	58 (77)	13 (81)
Hypertension (%)	32 (35)	23 (31)	9 (56)
Chronic kidney disease (%)	11 (12)	5 (7)	6 (38)
Chronic lung disease^a^ (%)	8 (9)	5 (7)	3 (19)
Stroke (%)	1 (1)	0	1 (6)
Cardiovascular disease (%)	3 (3)	3 (4)	0
Blood disorder^b^ (%)	2 (2)	2 (3)	0
Chronic liver disease (%)	2 (2)	2 (3)	0

a Chronic lung diseases include COPD and tuberculosis.

b Blood disorders include thrombocytopenia and thalassemia.

### Production of Recombinant Hcp1, TssM, LolC and AhpC

To obtain recombinant Hcp1, TssM, LolC and AhpC for use in the study of human immune responses, N-terminal His-tagged versions of these proteins were expressed and purified from *E. coli*. All proteins were extracted from whole-cell pellets in a soluble form and purified to homogeneity using tandem nickel-cobalt chromatography (28, 36). SDS-PAGE was used to assess the purity and structural integrity of the antigens (**Figure S1**). As expected, all of the proteins exhibited relative mobilities that were consistent with their predicted molecular weights. Endotoxin concentrations for the Hcp1 and TssM preparations were <1 EU/mg while those for the LolC and AhpC preparations were <5 EU/mg.

### Acute Melioidosis Patients Exhibit Strong IFN-*γ* Secreting T Cell Responses Against Hcp1 and TssM

**Figure 1:**
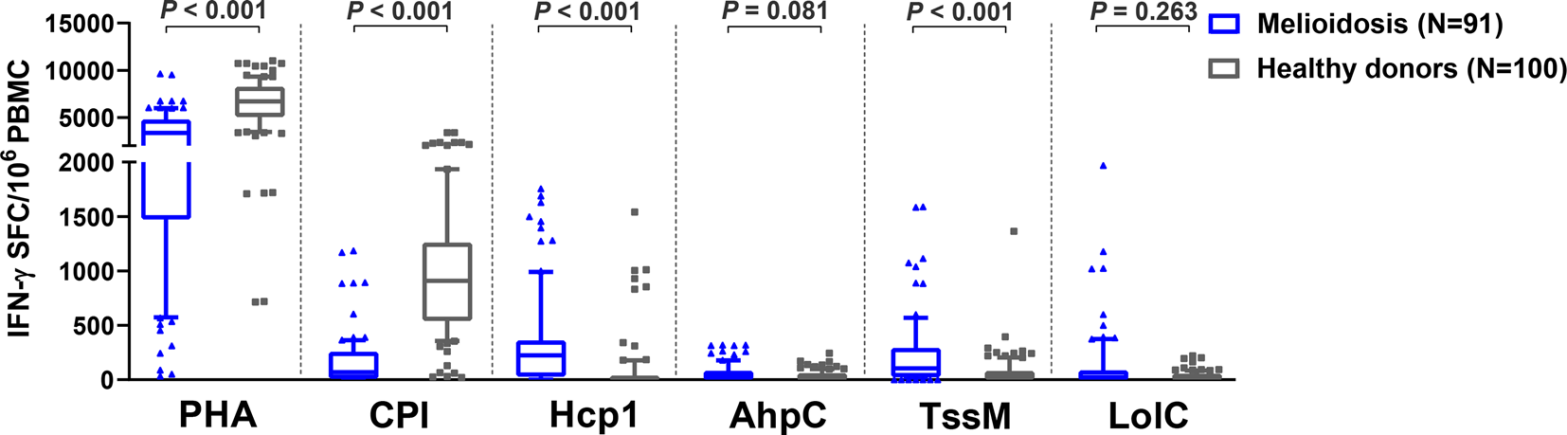
Cellular immune responses to different *B pseudomallei* vaccine candidate antigens in PBMC from patients during acute melioidosis (day 0) compared to healthy donors. PBMC from 91 melioidosis patients and 100 healthy donors were stimulated with recombinant *B pseudomallei* proteins (Hcp1, AhpC, TssM, LolC) and controls (PHA and CPI) for 24 hours *in vitro* and IFN-γ secreting cells were measured by ELISpot assays. Data displayed for each antigen is the number of spot forming cells (SFC) per 10^6^ PBMC. Box plots represent 25^th^ and 75^th^ percentile boundaries in the box with the median line within the box; the whiskers indicate the 10^th^ and 90^th^ percentiles. Statistical significance between groups was determined using the Mann-Whitney test.

Cellular immune responses to the vaccine candidate antigens were assessed in 91 patients with cultured-confirmed melioidosis cases and 100 healthy controls from a blood donation clinic at Mukdahan Hospital, Thailand. Cryopreserved PBMC from melioidosis patients and healthy donors were cultured with recombinant *B. pseudomallei* Hcp1, TssM, LolC, and AhpC and then analyzed for IFN-γ production by ELISpot assay. PHA and the CPI peptide pool were used as positive control antigens. Among *B. pseudomallei* antigens, cellular immune responses to Hcp1 and TssM *ex vivo* were significantly higher in melioidosis patients compared to healthy donors (**Figure 1**). The medians and interquartile ranges (IQR) of IFN-*γ* production of immune cells presented as SFC/10^6^ PBMC in melioidosis patients and healthy donors for all antigens were as follows: Hcp1, 225 (30–360) *versus* 5 (0–30), *P* < 0.001; AhpC 25 (0-80) *versus* 15 (0-58.75), *P* = 0.081; TssM 105 (25-290) *versus* 30 (0-78.75), *P* < 0.001; LolC, 23 (0-85) *versus* 20 (0-54.5), *P* = 0.263. Interestingly, acute melioidosis patients had significantly lower median levels of IFN-γ production from PBMC in response to PHA and CPI peptides compared to healthy donors [median (IQR) PHA, 3365 (1475-4754) *versus* 6703 (5073-8224), *P* < 0.001; CPI 69 (10-255) *versus* 910 (542-1259), *P* < 0.001].

### CD4+ T Cells Are the Predominant IFN-γ Secreting Cells Against Hcp1 and TssM in Acute Melioidosis Patients

**Figure 2:**
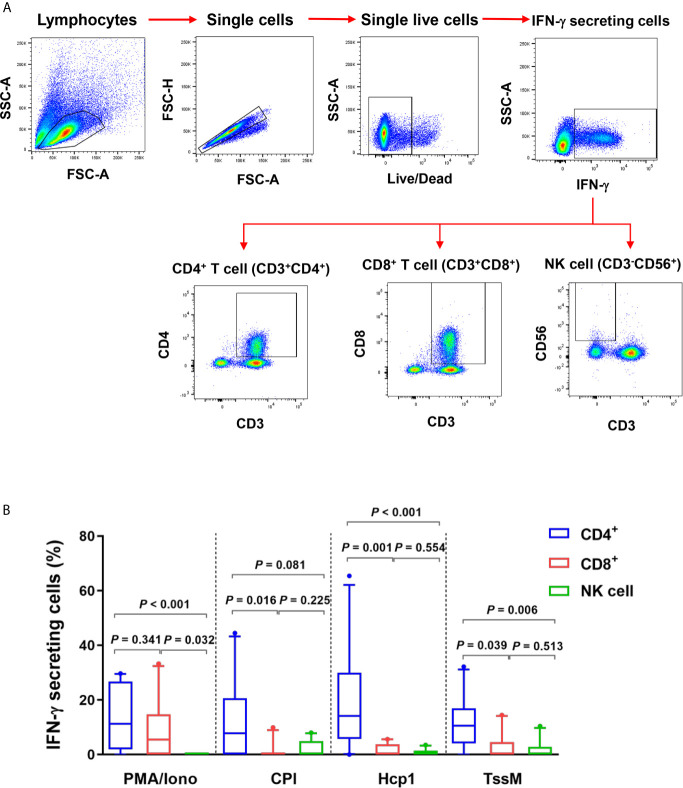
IFN-*γ* producing cells from acute melioidosis patients in response to *B pseudomallei* Hcp1 and TssM or positive controls [PMA plus ionomycin (PMA/Iono) and CPI]. **(A)** Flow cytometry gating strategy for IFN-γ secreting CD4^+^ T cells, CD8^+^ cells and NK cells. PBMC from one representative melioidosis patient were incubated with positive controls [PMA plus ionomycin (PMA/Iono)] and stained for intracellular IFN-*γ versus* the T cell markers; CD3, CD4, CD8 and NK cell marker; CD56. Frequencies of IFN-*γ* secreting CD4^+^ T cells, CD8^+^ T cells and NK cells were identified within total IFN-*γ* secreting cells. **(B)** PBMC from 10 melioidosis patients were stimulated with the proteins and controls for 6 hours and then stained for intracellular IFN-*γ*. Frequencies of CD4^+^ T cells, CD8^+^ T cells and NK cells within total IFN-γ producing cells are shown. Box plots represent 25^th^ and 75^th^ percentile boundaries in the box with the median line within the box; the whiskers indicate the 10^th^ and 90^th^ percentiles. Statistical significance between groups was determined using the Mann-Whitney test.

Since IFN-γ ELISpot assays showed that Hcp1 and TssM induced strong cellular responses in PBMC from melioidosis patients during acute infection, we next utilized flow cytometry to identify the source(s) of this cytokine. PBMC from 10 acute melioidosis patients were stimulated with Hcp1 and TssM and then analyzed for intracellular IFN-*γ* as well as for surface markers for CD4^+^ (CD3^+^CD4^+^) T cells, CD8^+^ (CD3^+^CD8^+^) T cells and NK (CD3^-^CD56^+^) cells. To accomplish this, lymphocytes were gated and the frequencies of IFN-*γ* secreting CD4^+^ T cells, CD8^+^ T cells and NK cells were determined (**Figure 2A**). Using this approach, we found that IFN-γ secreting cells in acute melioidosis patients were predominantly CD4^+^ T cells (**Figure 2B**). We also found that the frequency of these CD4^+^ T cells was significantly higher than the frequency of CD8^+^ T cells and NK cells secreting the same cytokine in response to stimulation with Hcp1 or TssM (P < 0.05 for all comparisons). Additionally, while PMA plus ionomycin was shown to stimulate robust production of IFN-*γ* from CD8^+^ T cells, only weak production was observed when the same type of cells was stimulated with Hcp1, TssM and CPI peptides. Furthermore, we observed a significantly lower frequency of IFN-*γ* producing NK cells in response to stimulation with PMA plus ionomycin compared to both CD4^+^ T cells (*P* < 0.001) and CD8^+^ T cells (*P* = 0.032) from melioidosis patients.

### Enhanced T Cell Responses Are Associated With Survival in Acute Melioidosis Patients

**Figure 3:**
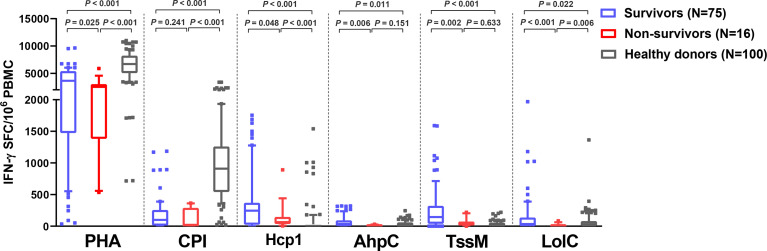
Cellular immune responses to *B pseudomallei* vaccine candidate antigens in melioidosis patients during acute infection in survivors and non-survivors. IFN-γ ELISpot assays were performed with cryopreserved PBMC from 100 healthy donors, 75 survivors and 16 non-survivors. Data displayed for each antigen is the number of spot forming cells (SFC) per 10^6^ PBMC. Box plots represent 25^th^ and 75^th^ percentile boundaries in the box with the median line within the box; the whiskers indicate the 10^th^ and 90^th^ percentiles. Statistical significance between groups was determined using the Mann-Whitney test.

Of 91 melioidosis patients, 16 (17%) died within 28 days of enrollment. Here, we investigated cellular immune responses relative to disease outcomes by comparing the IFN-γ ELISpot assay results using PBMC from 75 survivors and 16 non-survivors. The frequency of IFN-γ producing cells in response to all *B. pseudomallei* antigens were significantly higher in survivors compared with non-survivors (**Figure 3**). The medians and IQR of IFN-γ ELISpot assays presented as SFC/10^6^ PBMC of survivors and non-survivors were as follows: Hcp1, 245 (25-368) *versus* 64 (37-144), *P* = 0.048; AhpC, 35 (3-90) *versus* 7 (0-25), *P* = 0.006; TssM 145 (40-320) *versus* 38 (11-71), *P* = 0.002; LolC 35 (0-135) *versus* 0 (0-11), *P* < 0.001. For controls, we observed significantly lower IFN-*γ* responses with PHA in non-survivors compared to survivors but not with the CPI control [PHA, median (IQR) 2530 (1384-3015) *versus* 3650 (1475-5390), *P* = 0.025; CPI, 15 (7-288) *versus* 100 (10-255), *P* = 0.241]. Interestingly, IFN-γ secreting cells activated by the CPI peptides in melioidosis patients (both survivors and non-survivors) were significantly lower when compared with those of healthy donors with the median (IQR) of survivors, non-survivors and healthy donorsbeing 100 (10-255), 15 (7-288) and 910 (543-1259), respectively (*P* < 0.001 for healthy donors *versus* survivors and healthy donors *versus* non-survivors).

### Recovered Melioidosis Patients Exhibit High IFN-γ Secreting T Cell Responses Against Hcp1 and TssM

**Figure 4:**
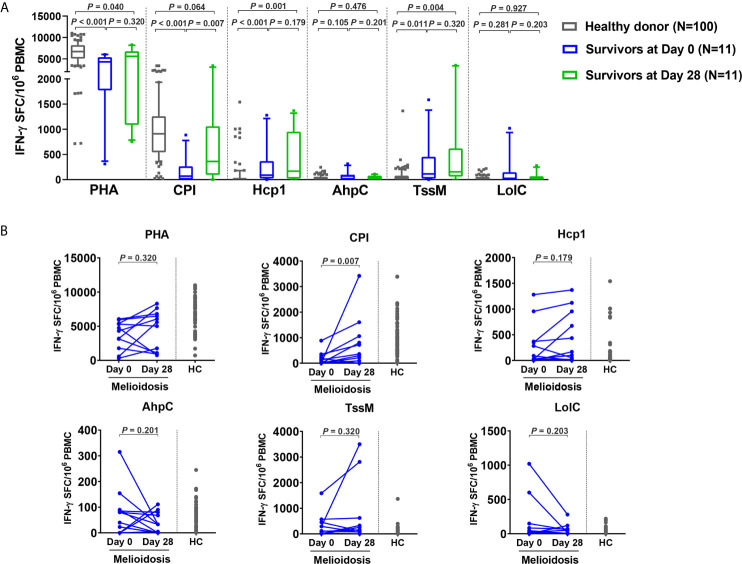
Cellular immune responses to *B pseudomallei* vaccine candidate antigens in survivors at day 28 versus day 0. **(A)** Antigen-specific IFN-γ secreting cells were analyzed in 11 survivors at day 0 and day 28 and in 100 healthy donors. Box plots represent 25^th^ and 75^th^ percentile boundaries in the box with the median line within the box; the whiskers indicate the 10^th^ and 90^th^ percentiles. Statistical significance between healthy donor and melioidosis patients was determined using the Mann-Whitney test and significant differences between time points within a group were determined using paired Wilcoxon test. **(B)** Antigen-specific IFN-γ secreting cells of melioidosis survivors at day 0 and day 28. Data from healthy donors (HC) were plotted as controls.

To assess the status of T cell activation in patients during recovery, specific T cell responses to different antigens were examined in 11 follow-up melioidosis patients who survived at day 28. PBMC were analyzed using IFN-γ ELISpot assays at day 0 and day 28 post-enrollment. When stimulated with AhpC and LolC, IFN-γ responses by cells obtained from melioidosis patients on day 0 and day 28 were low, and were not significantly different from the healthy donor controls (**Figure 4A**). In contrast, when stimulated with Hcp1 and TssM, the frequency of IFN-*γ* producing cells were higher at day 28 than at day 0, and were significantly increased compared to healthy donors. The medians (IQR) of healthy donors, survivors at day 0 and day 28 were as follows: Hcp1, healthy donors; 5 (0-30), survivors at day 0; 90 (23-368) and survivors at day 28; 168 (20-953) (healthy donors *versus* survivors at day 0, *P* < 0.001; healthy donors *versus* survivors at day 28, *P* = 0.001); TssM, 30 (0-79), 115 (20-453) and 155 (62-620) (healthy donors *versus* survivors at day 0, *P* = 0.011; healthy donors *versus* survivors at day 28, P = 0.004). Interestingly, cellular immune responses to the CPI peptides were significantly increased in recovered melioidosis patients at day 28 compared to day 0 [median (IQR) IFN-γ producing cells 69 (0-265) *versus* 360 (95-1060) SFC/10^6^ PBMC, *P* = 0.007] and the median level at day 28 was not significantly different from those of healthy donors [median (IQR), 910 (543-1259), *P* = 0.064].

We analyzed the dynamics of IFN-γ producing cells in individual patients and noted that 8/11 of the follow-up patients had decreased levels of IFN-γ producing cells for AhpC and LolC during recovery as assessed at day 28 (**Figure 4B**). In contrast, increased numbers of IFN-γ producing cells were observed at day 28 from 7/11 and 6/11 melioidosis patients in the cumulative responses to Hcp1 and TssM, respectively, with a median (IQR) of IFN-*γ* producing cells at day 28/day 0 of 1.9-fold higher for Hcp1 [350 (23-955) *versus* 673 (168-1120) SFC/10^6^ PBMC] and 2.4-fold higher for TssM [194 (7.5-823) *versus* 474 (232.3-2983) SFC/10^6^ PBMC].

### Acute Melioidosis Patients Exhibit Strong Antibody Responses Against Hcp1 and AhpC

**Figure 5:**
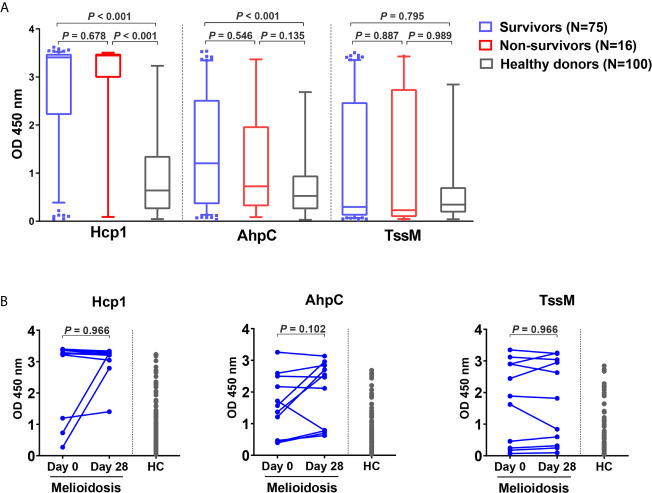
Antibody responses to Hcp1, AhpC and TssM in healthy donors and melioidosis patients. **(A)** IgG-specific responses are shown for acute melioidosis patients and healthy donors. Box plots represent 25^th^ and 75^th^ percentile boundaries in the box with the median line within the box; the whiskers indicate the 10^th^ and 90^th^ percentiles. P-values were calculated using the Mann-Whitney test. **(B)** IgG-specific responses of 11 melioidosis patients at day 0, day 28 and healthy donor controls (HC). Statistical significance between time points within a group were determined using the paired Wilcoxon test.

To investigate the humoral immune responses against the melioidosis vaccine candidate antigens and for comparison with cellular immune responses, we performed IgG ELISA with Hcp1, AhpC and TssM using plasma samples from 75 acute melioidosis survivors, 16 non-survivors and 100 healthy donors (**Figure 5A**). We did not include LolC in the IgG ELISA because the results from our pilot study showed a low level of antigen-specific IgG responses in plasma from melioidosis patients. The median optical density (OD) values of IgG responses to Hcp1 and AhpC for melioidosis survivors were significantly higher than those for healthy donors (*P* < 0.001 for all comparisons between survivors *versus* healthy donors). In contrast, IgG responses to TssM between the two melioidosis groups and healthy donors were not different. In survivors, the median OD value of IgG against Hcp1 was higher than those of AhpC and TssM [median (IQR) 3.4 (2.2 – 3.5), 1.2 (0.4 – 2.5) and 0.3 (0.1 – 2.5), respectively]. In healthy donors, the median OD (IQR) values of IgG against Hcp1, AhpC and TssM were low at 0.6 (0.2 – 1.3), 0.4 (0.2 – 0.8) and 0.3 (0.2 – 0.6), respectively. However, there were no significant differences between survivors and non-survivors for IgG responses to all *B. pseudomallei* antigens. We analyzed the relationship between the number of IFN-*γ* producing cells and IgG levels against these same antigens but did not observe high correlations (rho = 0.69 for Hcp1, 0.79 for AhpC and 0.13 for TssM) (**Figure S2**). Specific IgG responses were also examined in 11 convalescent patients who survived at day 28. In this group, we observed increased levels of IgG responses from 3/11, 7/11 and 6/11 melioidosis patients in response to Hcp1, AhpC and TssM, respectively (**Figure 5B**).

## Discussion

Understanding immune responses against *B. pseudomallei* antigens in patients during active infections is critical for vaccine development. This study characterized both cellular and humoral immune responses against four vaccine candidate antigens using PBMC and plasma from 91 melioidosis patients and 100 healthy donors living in Northeast Thailand. We observed that IFN-γ responses from PBMC from acute melioidosis patients, upon stimulation with Hcp1 and TssM, were significantly greater than in PBMC from healthy donors. The majority of IFN-γ secreting cells against Hcp1 and TssM in acute melioidosis patients were CD4^+^ T cells. During acute infection, survival was associated with T cells producing strong IFN-*γ* responses against all four of the *B. pseudomallei* antigens examined in this study. Enhanced cellular responses to Hcp1 and TssM were observed in survivors at day 28 compared to healthy donors. We also showed that antigen-specific IgG responses against Hcp1 and AhpC were higher in survivors compared to healthy donors.

*B. pseudomallei* Hcp1, AhpC, TssM and LolC are known to be highly immunogenic and protective antigens in animal models of melioidosis. Mice immunized with these proteins have also been shown to produce robust IFN-*γ* secreting T cell responses (11, 14, 15, 21). Melioidosis patients showed strong T cell responses against AhpC and LolC that were significantly higher than seronegative control subjects (21, 26, 27). However, we did not observe a significant difference between acute melioidosis patients and healthy donors when PBMC were stimulated with AhpC and LolC. This may be due to variation between individuals in their immune responses to *B. pseudomallei* antigens. Our study additionally demonstrated a high frequency of IFN-γ secreting cells in human PBMC against *B. pseudomallei* Hcp1 and TssM, and showed that the magnitude of the responses from acute melioidosis was significantly higher than in healthy donors. 6% of healthy controls exhibited strong cellular responses to Hcp1 and TssM. This may be a result of repeated environmental exposure to *B. pseudomallei* or successful clearance of *B. pseudomallei* after exposure (37, 38). In previous studies, there was no difference in IFN-γ production by PBMC from melioidosis patients *versus* healthy controls upon stimulation with cytomegalovirus, Epstein–Barr virus and influenza virus (CEF) peptide pool which is a positive control for CD8^+^ T cell functionality (26, 39). Our use of the CPI peptide pool, a positive control for the functionality of CD4^+^ T cells and antigen presenting cells (APC), demonstrated a significantly lower number of IFN-γ secreting T cells upon stimulation of PBMC from acute melioidosis patients compared to healthy donors. Kronsteiner et al. have reported that lower CD4^+^ T cell numbers were observed in acute melioidosis patients compared to healthy controls (40). Therefore, the lower T cell responses in acute melioidosis patients in our study may reflect suppression of CD4^+^ T cell expansion or a defect in antigen presentation and T cell activation processes during acute melioidosis.

It has been previously reported that CD4^+^, CD8^+^ and NK cells from melioidosis patients are sources of IFN-γ production following stimulation with *B. pseudomallei* proteins such as LolC, OppA and PotF or whole bacteria (27, 37, 39). Similar to our study, Rongkard et al. reported that acute melioidosis patients predominantly exhibited IFN-γ production from CD4^+^ T cells when stimulated with culture filtrate antigens from *B. pseudomallei* (37). CD4^+^ T cells, but not CD8^+^ T cells have also previously been shown to be important in mediating immunity against *B. pseudomallei* infections using depletion studies in a murine model (41). We observed low levels of IFN-γ producing CD8^+^ T cells against both Hcp1 and TssM in acute melioidosis patients. This may be due to a defect in specific immunity to *B. pseudomallei* during acute infection since no significant difference was seen in IFN-γ producing CD4^+^ T cells *versus* CD8^+^ T cell in response to positive controls. However, the percentage of IFN-γ producing CD8^+^ T cells specific for *B. pseudomallei* was significantly increased in recovered melioidosis patients (39). A previous study demonstrated low numbers of IFN-γ producing NK cells against heat-killed *B. pseudomallei* in acute melioidosis (39). This is consistent with our results that showed low cellular responses from NK cells during acute melioidosis. Additional studies showed that upon the stimulation of human PBMC from seropositive individuals, or those who had recovered melioidosis, that NK cells were transient and predominated during the first 24 hours while CD4^+^ and CD8^+^ T cells were more important in the later phases of infection (27).

Enhanced cellular immune responses have been previously observed in survivors of acute melioidosis (21, 26, 39). In this study, our findings showed that IFN-γ secreting T cell responses against all four *B. pseudomallei* proteins tested were significantly greater in survivors compared to non-survivors. This is consistent with previous studies showing that AhpC-specific responses were associated with survival in patients with acute melioidosis (21, 26), and Tippayawat et al. also reported strong T cell responses against LolC in recovered melioidosis patients and seropositive individuals. Our findings also support previous studies demonstrating that LolC, Hcp1 and TssM are protective antigens in mouse models of melioidosis (11, 15, 36).

Follow-up studies focused on cellular responses through one year after acute infections have been conducted in melioidosis patients (26, 39). In this study, IFN-γ responses in survivors at day 28 after acute melioidosis were consistent with the responses reported in previous studies examining T cell responses to *B. pseudomallei* (26, 27, 39). The frequency of IFN-γ producing cells against LolC and AhpC was significantly increased in recovered melioidosis compared to seronegative control subjects (26, 27). However, in this study, we only observed increased numbers of IFN-γ producing cells in PBMC from majority of recovered melioidosis patients at day 28 when they were stimulated with Hcp1 and TssM. In addition, we showed that CD4^+^ T cell responses against the CPI peptide pool were restored at day 28 after acute infection and demonstrated similar responses as healthy controls.

Serum antibodies against *B. pseudomallei* cell surface polysaccharides and proteins are detectable in acute melioidosis infections (26, 28, 42–44). There is some evidence in human and animal models that antibody responses play a key role in protection against melioidosis (14, 15, 26, 31, 45, 46). Our previous studies showed that melioidosis patients who lived in endemic areas had high IgG responses to Hcp1 and O-polysaccharide (28, 31, 47). This correlates with the data obtained in this study showing that human antibodies specific for Hcp1, AhpC and TssM can be detected in plasma. The levels of both IgG and IgM against heat-killed *B. pseudomallei* in survivors and non-survivors have been shown to be comparable (45). This is consistent with our study showing that there are no significant differences in IgG levels against *B. pseudomallei* proteins in survivors and non-survivors. Further studies are required to investigate potential role(s) for antibodies against Hcp1, AhpC and TssM. Interestingly, Chaichana et al. demonstrated that total IgG from survivors enhanced phagocytic activities and intracellular growth inhibition after phagocytosis (45) but they did not provide information about the target antigens of these antibodies.

There were some limitations to this study. We need larger sample sizes from those who die within 28 days as well as those who recover from melioidosis to confirm T cell responses against *B. pseudomallei* antigens and the CPI peptide pool. The samples from this study were collected from only one hospital from an endemic area in Northeast Thailand therefore this warrants further evaluation in other areas.

In conclusion, this study demonstrated that T cells from melioidosis patients recognized four candidate vaccine antigens of *B. pseudomallei* (Hcp1, AhpC, TssM and LolC) at different levels. In particular, strong IFN γ-secreting T cell responses to Hcp1 and TssM were associated with survival in patients. Melioidosis patients also had strong IgG responses to Hcp1 but no correlational relationship was identified between the number of IFN-γ producing cells and IgG levels. Collectively, these findings further support our rationale for using Hcp1 and TssM to develop subunit vaccines to combat disease caused by *B. pseudomallei*.

## Data Availability Statement

The raw data supporting the conclusions of this article will be made available by the authors, without undue reservation.

## Ethics Statement

Human studies and consent forms were approved by the Ethics committees of the Faculty of Tropical Medicine, Mahidol University (approval number MUTM 2015-002-005 and MUTM 2018-039-02), Mukdahan Hospital (approval number MEC 10/59 and MEC 07/61) and the U.S. Army Medical Research and Development Command, Office of Research Protections, Human Research Protection Office (approval numbers A-20848.a and A-20848.b). The study was conducted according to the principles of the Declaration of Helsinki (2008) and the International Conference on Harmonization (ICH) Good Clinical Practice (GCP) guidelines. Written informed consent was obtained for all patients and healthy donors enrolled in the study. The patients/participants provided their written informed consent to participate in this study.

## Author Contributions

PB, MB, and NC conceived and designed the experiments. AY, RJ, SS, OO, and CM collected clinical samples. SS performed the experiments and analyzed the data. PB, MB, and LS contributed reagents and recombinant protein antigens. SS, NC, EW, PB, and MB wrote the manuscript. All authors contributed to the article and approved the submitted version.

## Funding

This research was supported by Defense Threat Reduction Agency contract HDTRA1-18-C-0062, Medical CBRN Defense Consortium contract MCDC-18-04-11-004 and the National Institute of Allergy and Infectious Diseases of the National Institutes of Health (NIH/NIAID), Grant No. U01AI115520). The content is solely the responsibility of the authors and does not necessarily represent the official views of the funders.

## Conflict of Interest

The authors declare that the research was conducted in the absence of any commercial or financial relationships that could be construed as a potential conflict of interest.

## Publisher’s Note

All claims expressed in this article are solely those of the authors and do not necessarily represent those of their affiliated organizations, or those of the publisher, the editors and the reviewers. Any product that may be evaluated in this article, or claim that may be made by its manufacturer, is not guaranteed or endorsed by the publisher.
