# Supplementary material for: Melioidosis Patient Survival Correlates With Strong IFN-γ Secreting T Cell Responses Against Hcp1 and TssM
Source: Front Immunol. 2021 Jul 30;12:698303. doi: 10.3389/fimmu.2021.698303 (PMC8363298; doi:10.3389/fimmu.2021.698303)
Supplement: Supplementary file 1 [file DataSheet_1.pdf]

SUPPLEMENTARY MATERIAL

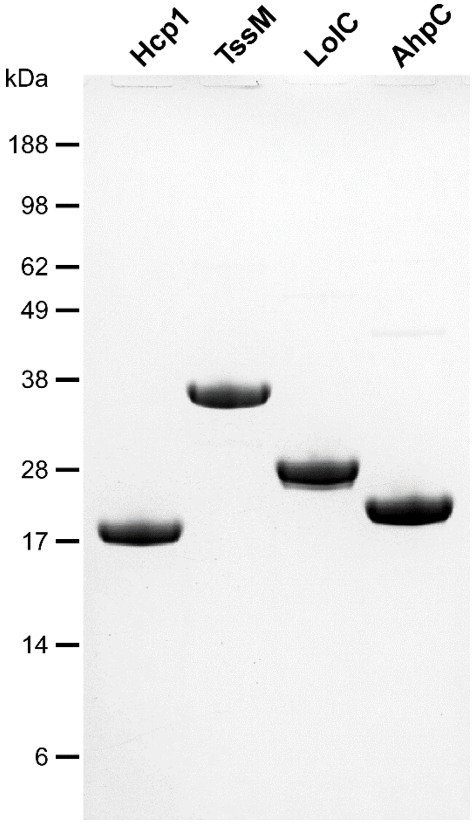

FIGURE S1. SDS-PAGE analysis of purified recombinant proteins (Hcp1, TssM, LolC and AhpC). The purified proteins were separated on a 12 % Bis-Tris gel and visualized using SimplyBlue Safe Stain. Approximately 3 µg of each protein was loaded onto the gel. The protein molecular standards (kDa) are indicated on the left.

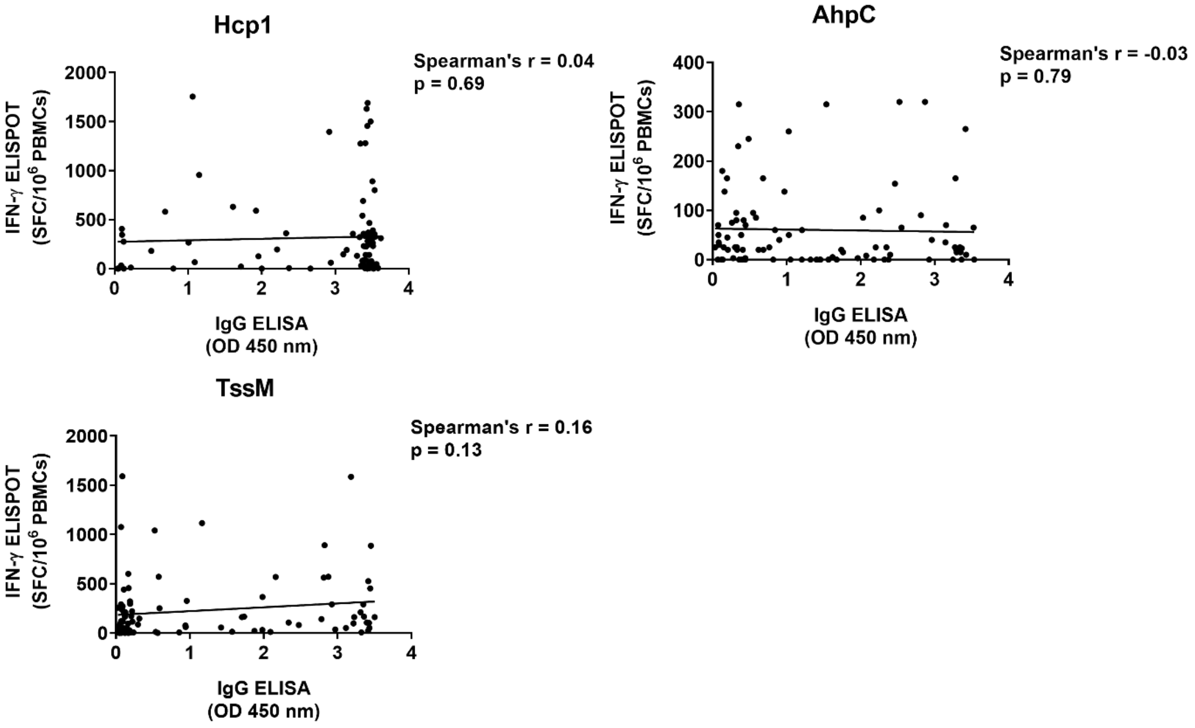

FIGURE S2. Relationship between cellular responses to *B. pseudomallei* antigens measured by IFN-γ ELISpot assays and levels of specific IgGs measured by ELISA (n=91). Scatter plots with linear regression lines are presented with Spearman's R.

TABLE S1. Oligonucleotide primers used in this study.

Primer	Primer Sequence[a]	Reference
Bmhcp1-6HisF	5'-CCCAACGGTCTCACATGGCGGCGCATCATCATCATCATCATCTGGCCGGAATATATCTCAAGG-3'	1
Bmhcp1-R1	5'-CCCAACGGTCTCAAGCTTCAGCCATTCGTCCAGTTTGCGGC-3'	1
BpahpC-FHis1	5'-CCCAACCGTCTCCCATGGCGGCGCATCATCATCATCATCATAAGACCGTGGGCGATAAACTCGAAG-3'	This study
BpahpC-R1	5'-CCCAACCGTCTCTAGCTTTACAGCGTCGCGCCGCCGATC-3'	This study

[a] The underlined sequence are regions cleaved by restriction endonucleases.

Reference

1. Pumpuang A, Dunachie SJ, Phokrai P, Jenjaroen K, Sintiprungrat K, Boonsilp S, et al. Comparison of O-Polysaccharide and Hemolysin Co-Regulated Protein as Target Antigens for Serodiagnosis of Melioidosis. PLoS Negl Trop Dis (2017) 11(3):e0005499. doi: 10.1371/journal.pntd.0005499
